# Body Balance and Physiotherapy in the Aquatic Environment and at a Gym

**DOI:** 10.1155/2021/9925802

**Published:** 2021-06-21

**Authors:** Magdalena Pieniążek, Grzegorz Mańko, Michał Spieszny, Jan Bilski, Wojciech Kurzydło, Tadeusz Ambroży, Jarosław Jaszczur-Nowicki

**Affiliations:** ^1^Department of Rehabilitation in Internal Diseases, Faculty of Rehabilitation, Bronisław Czech University School of Physical Education in Kraków, Al. Jana Pawła II 78, 31-571 Kraków, Poland; ^2^Faculty of Health Sciences, Department of Biomechanics and Kinesiology, Medical College of the Jagiellonian University in Kraków, Grzegórzecka 20, 31-531 Kraków, Poland; ^3^ORNR “Krzeszowice” Rehabilitation Centre, Daszyńskiego 1, 32-065 Krzeszowice, Poland; ^4^Institute of Sport Sciences, Faculty of Physical Education and Sports, University of Physical Education, Aal. Jana Pawła II 78, 31-571 Kraków, Poland; ^5^Faculty of Health Sciences, Clinic of Rehabilitation, Medical College of the Jagiellonian University in Kraków, Michałowskiego 12, 31-126 Kraków, Poland; ^6^Faculty of Physical Education and Sport, University of Physical Education, Al. Jana Pawła II 78, 31-571 Kraków, Poland; ^7^Department of Tourism, Recreation and Ecology, University of Warmia and Mazury in Olsztyn, Oczapowskiego 5, 10-719 Olsztyn, Poland

## Abstract

The increase in the average age of our society represents a growing medical and social problem, which requires concentration on the issue concerning balance disorders. The aquatic environment has a number of complex properties that have miscellaneous effects on the human body. In the light of the above, water is becoming an ideal environment to learn correct neuromuscular communication, and a properly prepared training session in water helps to practice balance and movement coordination. The objective of the study was to assess the impact of rehabilitation in the aquatic environment on patients' balance and compare the results obtained with patients who received rehabilitation at a gym. The study was carried out among patients hospitalised in the “Krzeszowice” Rehabilitation Centre. It encompassed 137 patients, randomly assigned to either the study group (the aquatic environment) or the control group (the gym). The preliminary examination included general medical history and a test on the stabilometric platform. The patients attended training sessions for 4 weeks, 5 times a week for 30 minutes. It was a single-blinded study wherein the authors did not know which group a given patient had been assigned to. Upon completion of a monthly therapy, the stabilometric test was carried out again. The study revealed that the patients from both groups experienced a significant improvement in balance. However, the improvement was slightly greater in those exercising in the pool. Physiotherapy in the aquatic environment makes a greater contribution to the improvement of body balance compared to physical exercises performed at a gym.

## 1. Introduction

The increase in the average age of our society [1] represents a growing medical and social problem, as well as requires concentration on the issue concerning balance disorders, also connected with common chronic spine problems [2]. Deep sensation, also termed proprioception, is a sensory function which allows us to precisely and consciously determine the position of our body in space. As a result, we are able—without visual control—to identify the movement of one body part against the other, as well as define the position of our limb and the degree of muscle tension or stretching. Thanks to the receptors of deep sensation, the force of muscle contraction is adequate for the increased reflexive muscle strain. They send information to which the body responds with appropriate tension or a quick change in muscle length. Depending on the type of receptors, they are stimulated from the initial to the final stage of movement while increasing the speed and direction of movement, or they are related to feeling the position of muscles and joints, ensuring a proper sequence of tissue contraction, stabilisation of adequate joints, and protection of a muscle against damage due to excessive stretching. These receptors make it possible to perform even very complex and quick movements [3].

The favourable conditions of the aquatic environment allow for producing multidimensional and multifaceted effects on the patient. The therapeutic effects on the human body result from the specific properties of the aquatic environment, including the hydrostatic pressure, viscosity, upthrust, and the thermal factor. A very important mechanical factor was the phenomenon of buoyancy (upthrust). According to Archimedes' principle, the buoyancy force acts vertically upwards, opposite to the force of gravity. Consequently, the resultant forces acting on the human body immersed in water are smaller from its weight on the ground and dependent on the depth of immersion. Weakened muscles may undergo an effective exercise session with considerably lower strain [4]. The buoyancy of water minimises the incidence of injuries during exercise, thereby making the patients feel more confident and secure despite the more challenging environment [5]. The aquatic environment was already described over two decades ago as the one that induces increased afferent stimulation caused by the skin-water contact. As a result, patients are less fearful and more confident of their movements, and activity in water may facilitate vestibular inputs [6].

A properly prepared training session in water helps to practice balance and movement coordination. Exercising in deep water with various types of flotation devices (buoyancy aids) that increase the state of imbalance is aimed at finding the right solutions for perfect movements. Learning the right body positioning and the awareness of the movements performed and the tension and relaxation of the body, as well as the maximum use of one's own capacities, practice deep sensation in water, which practically cannot be practiced in such an intensive and, at the same time, safe manner at a gym [3]. At the same time, it should be borne in mind that, at this age, the problem with maintaining a stable body posture is complex and often leads to falls, which may result in severe injuries or even death. The issue that also needs attention is the huge costs generated by falls [7].

The main hypothesis was that the impact of physiotherapy in the aquatic environment on the patient's body balance is greater than that in patients undergoing training at the gym. The main reason for conducting this study was the increase in the average age of our society, which in the near future will constitute a growing medical and social problem. It will require the attention of the medical community due to its health and economic consequences.

## 2. Material and Methods

The study was conducted from June 10, 2017, to June 12, 2018, among patients from the “Krzeszowice” Rehabilitation Centre, who had been prescribed a 4-week rehabilitation stay for general health improvement. 151 patients meeting the inclusion criteria were qualified for the study. Out of this number, 137 patients expressed their consent to participate in the study. By using a special program, they were randomly assigned to the study group (73 participants) and the control group (64 participants). The patients were aged from 32 to 88 years, with the average age being 63.53 years ± 10.92 years. The study group consisted of 73 patients, including 40 men (54.79%) and 33 women (45.21%), while the control group consisted of 64 patients, including 31 men (48.44%) and 33 women (51.56%). The most common concomitant diseases were hypertension (in 49% of patients), osteoarthritis/discopathy (in 30% of patients), and diabetes (in 20% of patients).

Inclusion criteria were written consent of the patient to participate in the study, no contraindications to undertake the proposed forms of physical activity, and patient age ≥ 30 years.

Exclusion criteria were symptomatic coronary artery disease, exertional dyspnoea, resting blood pressure higher than 160/100 mmHg, resting tachycardia higher than 100/min, persons with severe visual impairment, use of orthopedic equipment, vestibular disorders, hydrophobia, exercise asthma, circulatory-respiratory failure (from NYHA III), musculoskeletal dysfunction preventing the taking of the proposed forms of physical activity, hip/knee arthroplasty, stroke condition, Parkinson's disease, and other neurological diseases.

Patients were asked to determine whether they were physically active before arriving at the centre. Due to the lack of people who perform physical activity in accordance with WHO recommendations, patients were divided into regular and unsystematic exercisers. 13 people declared regular physical activity, performed at least once a week. Sports such as walking, nordic walking, running, and fitness have been listed. Occasional physical activity (performed less than once a week) was reported by 57 people, with 12 people mentioning work related to gardening, which they do not perform during the winter and late autumn. Other people mentioned walking, swimming, walking in the mountains, bicycle, and gymnastics. Almost half of the respondents (67 people) declared not engaging in any form of physical activity.

The patients attended a physiotherapy program in a swimming pool (the study group) and at a gym (the control group) 5 times a week for 30 minutes. The water temperature in the swimming pool was approx. 30-32°C, with an approximate temperature of 25-26°C at the gym. The exercises performed were aimed at overall functional improvement, with emphasis on gluteal, abdominal, dorsal, quadrilateral, gastrocnemius, and anterior tibial muscles. All of the patients were also scheduled for physiotherapy procedures (laser treatment, cryotherapy, and magnetic field treatment). It was a single-blinded study wherein the authors did not know which patient belongs to the study group and which to the control group.

Before and after the training session, the patients had their body balance checked on the FDM-S Zebris stabilometric platform (Zebris Medical GmbH, Germany). Each patient was asked to maintain an upright position for 30 seconds without any movement on a stabilometric platform. The study was carried out under three different conditions (with open eyes, closed eyes, and after 3 turns around one's own axis). Posture stability was assessed by analysing the body's centre of pressure (COP) according to the following four parameters:

- CEA—confidence ellipse area (the surface area covered by the COP pathway delineated during the study) expressed in square millimeter
- TTL—total trajectory length (the length of the pathway covered by the feet's centre of pressure within 30 seconds) expressed in millimeter
- HD—horizontal deviation (the average lateral deviation of the feet's COP from point 0, which is a geometric centre of gravity calculated for a given patient) expressed in millimeter
- VD—vertical deviation (the average anteroposterior deviation of the feet's COP from point 0) expressed in millimeter

The project was approved by the Bioethics Committee of the Jagiellonian University (number 122.6120.342.2016, 28 April 2017). A positive opinion was also obtained from the Scientific Research Team of the “Krzeszowice” Rehabilitation Centre (number 2/2017, 29 March 2017).

The paper made use of the Statistica 13.1 software, as well as the Shapiro-Wilk and chi-square tests. The analyses were conducted as double- and triple-factor analyses of variance (ANOVA).

## 3. Results

During the project, 7 individuals were excluded due to premature departure from the centre (without completing the 4-week training program) and common cold, which prevented them from participating in the training program for a few days. In the end, the project included 130 patients. No statistically significant differences were revealed between the study and the control groups in terms of demographic data (Table 1).

### 3.1. Analysis of Changes in Body Balance Parameters in All Patients

The stabilometric platform test was used to analyse the confidence ellipse area (the surface area covered by the COP pathway delineated during the study). The test was carried out twice (at the beginning and at the end of the project). A statistically significant (*p* < 0.001) improvement in the CEA, TTL, HD, and VD parameters was observed in all the subjects (Table 2).

### 3.2. Analysis of Changes in Body Balance Parameters in All Patients, including Study Conditions

In the case of result analysis taking into account three study conditions, the overall change was statistically significant. All body balance parameters delineated during the study decreased after the end of the training session (Table 3).

### 3.3. Analysis of Changes in Body Balance Parameters, including the Study and Control Groups

#### 3.3.1. CEA: Confidence Ellipse Area

**Figure 1:**
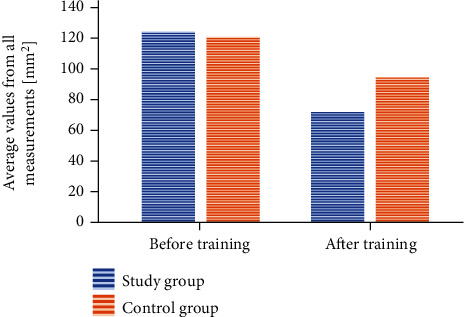
A change within the CEA value after the training session taking into account the particular groups.

The values measured in the study group before rehabilitation were 125.04 ± 65.6 mm^2^, while after physiotherapy,72.25 ± 53.1 mm^2^ (*p* < 0.001). The values measured in the control group in the beginning were 121.6 ± 65.6 mm^2^, while in the end,95.23 ± 53.1 mm^2^ (*p* < 0.001) (Figure 1). The difference between the average values was 52.79 mm^2^ in the group exercising in the pool and 26.36 mm^2^ in the control group.

The analysis of variance showed no statistically significant differences between the changes in the particular study conditions and the study and control groups (*p* < 0.785). However, it should be emphasized that the differences between the average values in each of the study conditions were slightly greater in the group exercising in the pool. In the case of the “open eyes” condition, the difference between the average values in the study group was 41.57 mm^2^, whereas in the control group, 16.61 mm^2^. Under the “closed eyes” condition, the values were 52.52 mm^2^ and 29.91 mm^2^, respectively, while after the turns 64.28 mm^2^ and 32.56 mm^2^.

#### 3.3.2. TTL: Total Trajectory Length

**Figure 2:**
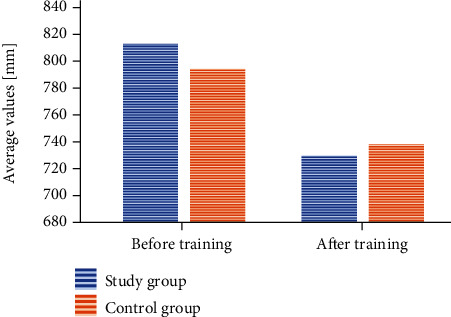
A change within the TTL value after the training session taking into account the particular groups.

The values measured in the study group before rehabilitation were 813.74 ± 186.8 mm, while after physiotherapy,730.04 ± 181.9 mm (*p* < 0.001). The values measured in the control group in the beginning were 794.92 ± 187.2 mm, while in the end,738.40 ± 182 mm (*p* < 0.001). The change was statistically significant in both groups, where its value was similar (Figure 2). The difference between the average values was 83.70 mm in the group exercising in the pool and 56.51 mm in the control group.

The analysis of variance showed no statistically significant differences between the changes in the particular study conditions and the study and control groups (*p* < 0.876). However, it should be emphasized that the differences between the average values in each of the study conditions were slightly greater in the group exercising in the pool. In the case of the “open eyes” condition, the difference between the average values in the study group was 68.58 mm, whereas in the control group, 51.36 mm. Under the “closed eyes” condition, the values were 96.36 mm and 67.28 mm, respectively, while after the turns—86.17 mm and 50.88 mm.

#### 3.3.3. HD: Horizontal Deviation

**Figure 3:**
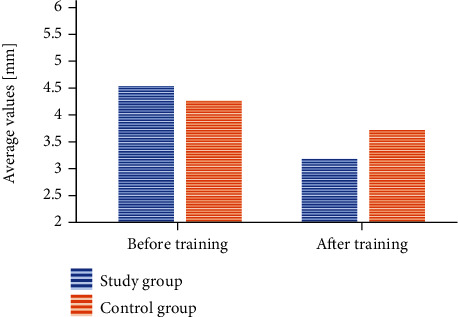
A change within the HD value after the training session taking into account the particular groups.

The values measured in the study group before rehabilitation were 4.55 ± 1.7 mm, while after physiotherapy,3.19 ± 1.4 mm (*p* < 0.001). The values measured in the control group in the beginning were 4.28 ± 1.7 mm, while in the end,3.73 ± 1.4 mm (*p* < 0.001). The change was statistically significant in both groups, but greater in the group exercising in the swimming pool (*p* < 0.001) (Figure 3). The difference between the average values was 1.36 mm in the group exercising in the pool and 0.56 mm in the control group.

The analysis of variance showed no statistically significant differences between the changes in the particular study conditions and the study and control groups (*p* < 0.365). Here, it is also worth emphasizing that the differences between the average values in each of the study conditions were slightly greater in the group exercising in the pool. Under the “open eyes” condition, the difference between the average values in the study group was 1.56 mm, whereas in the control group, 0.42 mm. Under the “closed eyes” condition, the values were 1.12 mm and 0.55 mm, respectively, while after the turns, 1.41 mm and 0.71 mm.

#### 3.3.4. VD: Vertical Deviation

**Figure 4:**
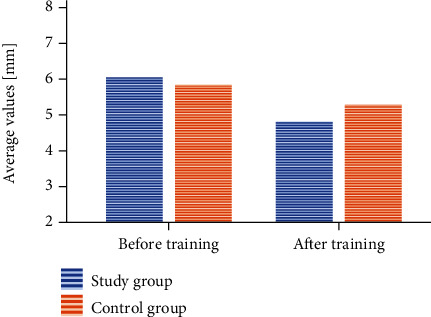
A change within the VD value after the training session taking into account the particular groups.

The values measured in the study group before rehabilitation were 6.07 ± 1.6 mm, while after physiotherapy, 4.83 ± 1.7 mm (*p* < 0.001). The values measured in the control group in the beginning were 5.87 ± 1.7 mm, while in the end, 5.30 ± 1.7 mm (*p* < 0.005). The change was statistically significant in both groups, but greater in the group exercising in the swimming pool (*p* < 0.017). The difference between the average values was 1.24 mm in the group exercising in the pool and 0.57 mm in the control group (Figure 4).

The analysis of variance showed no statistically significant differences between the changes in the particular study conditions and the study and control groups (*p* < 0.519). This is yet another result which demonstrated statistically significant changes in both groups. However, the differences between the average values in each of the study conditions were slightly greater in the group exercising in the pool. In the case of the “open eyes” condition, the difference between the average values in the study group was 0.83 mm, whereas in the control group, 0.45 mm. Under the “closed eyes” condition, the values were 1.49 mm and 0.79 mm, respectively, while after the turns, 1.39 mm and 0.47 mm.

### 3.4. Correlation between Change in Body Balance and Physical Activity before Arrival to the Centre, BMI, and Age

There was no correlation between parameters of body balance and physical activity, BMI, or age in the study group. In the control group, physical activity was positively correlated with the size of the change in TTL open eyes. BMI was negatively correlated with the size of the change in TTL after turns (Table 4).

No correlation between gender and change in body balance state was found.

An analysis was made regarding the impact of the age of the patients studied on their initial body balance. Patients were divided into two age groups—up to the age of 50 and over 50 [8]. A significant difference was observed only in the case of two parameters (HD open eyes, which was lower in patients ≤ 50 years, and TTL after the turns, which was lower in patients ≥ 50 years). Other measurements were similar. It is important to emphasize the large group discrepancy—over 90% of patients were over 50 years old.

## 4. Discussion

The review included literature from the following databases: PEDro, PubMed, Medline, Embase, and Scopus. The authors found no study that would use the same methodology. The discussion focuses on articles which are as close as possible to the authors' original project presented above. Our own study revealed that the patients participating in the project, in both the study and the control groups, manifested significant improvement in terms of balance. However, it is worth emphasizing that the patients exercising in the pool demonstrated a slightly greater improvement. Similar results were presented in the article by Simmons and Hansen, which compared the impact of exercise in the aquatic environment and at a gym on balance in elderly patients (average age over 80 years) [9]. Another study was conducted in patients with partial dysfunction of the vestibular system. Twenty-one patients with chronic vertigo (dizziness) received 10 physiotherapy sessions in water. The scale of dizziness and balance was checked using a balance platform before and after the rehabilitation therapy. The authors demonstrated the improvement of the patients' quality of life, decreased intensity of dizziness, and improved body balance [10]. The objective of the study by Booth was to compare the impact of exercise at a gym and in the aquatic environment on gait and balance in two groups of females aged 65 and more. Gait and balance were assessed using the Tinetti test. The results demonstrate that aquatic exercise may positively influence the gait and balance of the female subjects. However, no significant differences were found between the two groups. The author suggests that both types of exercise may be equally beneficial for the improvement of gait and balance. Exercise in the aquatic environment may be regarded as an alternative type of physical activity for seniors, especially if a land-based therapy is difficult to implement due to their chronic musculoskeletal conditions [11].

Another study was aimed at assessing the impact of an exercise program in the aquatic environment on daily routines. Sixty-six females (aged 60-89 years) were divided into two groups without randomisation, namely, the group exercising in water (*n* = 48) and the control group (*n* = 18). The subjects themselves chose the group, which was described by the authors as a limitation of the study. The training session encompassed a 16-week, supervised program based on the SWEAT™ method, carried out in shallow water (1.0-1.2 m), with water temperature of approx. 28-29° C. Compared with the control group, the patients exercising in water improved the parameters concerning their daily routines (e.g., the walking speed, the length of a step, agility, climbing up the stairs, and static balance). No change was observed in the dynamic balance. The results indicate that the SWEAT™ method applied to this program of aquatic exercise provides a safe and effective set of activities which can help elderly women to improve the management of their daily routines and static balance [12]. The results of another study comparing aquatic exercise with land-based exercise in patients with peripheral neuropathy demonstrate that physiotherapy in the aquatic environment revealed effects—comparable to those of the gym-based rehabilitation—on gait and balance dysfunctions. However, the Dynamic Gait Index was significantly more improved in the patients exercising in the aquatic environment [13]. The authors of the study aimed to examine the impact of hydrotherapy on gait and balance in poststroke patients. A single-blind, randomised, pilot study was carried out at an outpatient rehabilitation clinic of a neurological hospital in China. It encompassed a total of 28 participants (more than 6 months after stroke) with impaired gait and balance control. After initial assessment, the participants were randomly assigned to a land-based (control group, *n* = 14) or a hydrotherapy program (study group, *n* = 14). The participants underwent individual sessions for four weeks, five days a week, for 45 minutes per session. After four weeks of rehabilitation, all the participants were assessed by a “blinded” person. Functional assessments included the Functional Reach Test, the Berg Balance Scale, the 2-minute marching test, and the Timed Up and Go Test. After four weeks of treatment, the Berg Balance Scale, the results of the Functional Reach Test, the 2-minute marching test, and the Timed Up and Go Test improved significantly in each group (*p* < 0.05). The average improvement in the Functional Reach Test and the 2-minute marching test was significantly higher in the group exercising in water than in the control group (*p* < 0.01). This is yet another result consistent with our own results, in which both groups significantly recorded significant improvement in balance. However, those patients exercising in the aquatic environment tend to show greater improvement [14]. A positive impact of exercise in the aquatic environment on body balance was also presented in the study by Avelar et al. on overweight individuals [15].

The author's study attempted to assess the change in the balance obtained after training in relation to physical activity performed by patients before arriving at the rehabilitation centre. The results obtained indicate no relationship between these variables, correlation occurred only in the case of the control group and one balance parameter (TTL open eyes). However, due to the fact that only 13 people indicated that they exercise regularly (at least once a week), this number is too small to suggest some conclusions on this basis. It should also be emphasized that for preventive purposes, physical activity is recommended at least 3 times a week [16].

The study showed a correlation between BMI and change in balance parameter (TTL) in the control group. The lower the weight of the patient, the greater the change in balance. Perhaps, this is due to easier exercise at a gym for people who have less weight compared to those who are overweight/obese. The lack of a similar result in the case of a group exercising in the pool may indicate that greater body weight does not impede the exercise in the aquatic environment.

Gender had no effect on changes in body balance in our own study. The results of other studies that assess gender and balance usually do not show a statistically significant difference between women and men [17, 18]. However, according to Nolan et al., sexual dimorphism may be important for processes controlling body posture, especially at puberty [19]. This phenomenon in people is reflected in the morphological, physiological, and mental diversity, in women and men.

Obtained results regarding age and changes in balance do not give grounds to conclude that in our study age had an impact on the difference in body balance. Age also had no effect on baseline balance in the studied patients—significant changes were observed only in two parameters (HD open eyes, TTL after the turns). This is in contradiction with the results of other authors' studies [20, 21]. It should be emphasized, however, that in the author's study, the average age was around 65 years. Absolutely, age plays an important role in the stability of the body balance system, in the case of older people, over the years, there is a loss of muscle mass and strength, changes in the innervation of muscle fibers, and a decrease in conduction velocity of afferent and efferent stimuli, as well as a decrease in bone mass. Due to the fact that around the age of 30, muscle mass begins to decrease, but up to the age of 50, this is not very noticeable, perhaps this is the reason why the above result was obtained. The vast majority of patients (over 90%) were over 50 [8].

The literature review made by Howe et al. stresses that, despite numerous studies on exercises that improve balance in the elderly, there is still no reliable evidence that would confirm the effectiveness of a given training session [22]. This might be due to the commonly used, subjective, general, and imprecise methods of balance examination, which are not suitable for assessing the degree of disturbances. Tests that produce measurable diagnostic results have a much greater scientific value. Objective methods include posturographic tests, which make it possible to obtain a graphical record of the shifts in the centre of pressure (COP) [23]. The balance platform is a trusted and recognised method for assessing posture stability, increasingly used by researchers [24, 25]. Therefore, this method was applied to our own study.

The limitation of our own study was the manner in which the patients did the three turns during the balance test. Each of the subjects was asked to do 3 quick turns, so that s/he starts to feel slightly dizzy. The turns, therefore, were not performed identically by each subject. Moreover, the medications used by the patients were not taken into account (certain pharmaceuticals may affect patients' balance), while—prior to the study—the patients were asked whether they tended to feel dizzy. The study included only those individuals who manifested no balance problems.

Numerous studies indicate the positive impact of the aquatic environment on posture stability and the better effects than those achieved through standard exercises. It may also be a form of recreation that prevents and minimises the consequences emerging with age. A large percentage of people with arterial hypertension and degenerative changes of the spine with a simultaneous lack or little, regular physical activity among the studied patients emphasizes the need for a broader educational and preventive action.

## 5. Conclusions

The study conducted makes it possible to conclude that physiotherapy in the aquatic environment makes a greater contribution to the improvement of patients' body balance compared to physical exercises carried out at a gym.

## Figures and Tables

**Table 1:** Demographics.

Variables	Study group (*n* = 73)	Control group (*n* = 64)	*p*
Age (years)	62.58 ± 11.1	64.59 ± 10.69	0.28
Sex (M/W)	40/33	31/33	0.45
BMI (kg/m^2^)	28.27 ± 4.49	29 ± 4.01	0.32

*n*: number of patients; *p*: significance level; M: men; W: women; BMI: body mass index. Data are expressed as the arithmetic mean and standard deviation.

**Table 2:** Body balance parameters in all examined patients.

Variables		Mean ± sd	*p*
CEA (mm^2^)	Measurement 1	123.32 ± 66.3	<0.001
	Measurement 2	83.74 ± 53.81	
TTL (mm)	Measurement 1	804.33 ± 187.5	<0.001
	Measurement 2	734.22 ± 182.4	
HD (mm)	Measurement 1	4.42 ± 1.7	<0.001
	Measurement 2	3.46 ± 1.4	
VD (mm)	Measurement 1	5.97 ± 1.7	<0.001
	Measurement 2	5.07 ± 1.7	

CEA: confidence ellipse area; TTL: total trajectory length; HD: horizontal deviation; VD: vertical deviation; *p*: significance level. Data are expressed as the arithmetic mean and standard deviation.

**Table 3:** Analysis of changes in body balance parameters in all patients, including study conditions.

Study conditions	Variables		Mean ± sd	*p*
Open eyes	CEA (mm^2^)	Measurement 1	88.5 ± 53.3	<0.001
		Measurement 2	59.41 ± 43.6	
	TTL (mm)	Measurement 1	742.38 ± 190.5	<0.001
		Measurement 2	682.41 ± 186.1	
	HD (mm)	Measurement 1	3.96 ± 1.8	<0.001
		Measurement 2	2.97 ± 1.5	
	VD (mm)	Measurement 1	4.94 ± 1.8	<0.001
		Measurement 2	4.30 ± 1.8	
Closed eyes	CEA (mm^2^)	Measurement 1	151.33 ± 83.6	<0.001
		Measurement 2	110.12 ± 80.4	
	TTL (mm)	Measurement 1	886.67 ± 249.1	<0.001
		Measurement 2	804.85 ± 196.3	
	HD (mm)	Measurement 1	4.56 ± 2.1	<0.001
		Measurement 2	3.73 ± 1.8	
	VD (mm)	Measurement 1	7.47 ± 2.6	<0.001
		Measurement 2	6.33 ± 2.6	
After the turns	CEA (mm^2^)	Measurement 1	130.12 ± 100.3	<0.001
		Measurement 2	81.70 ± 66.5	
	TTL (mm)	Measurement 1	783.93 ± 220.1	<0.001
		Measurement 2	715.41 ± 230.9	
	HD (mm)	Measurement 1	4.73 ± 2.2	<0.001
		Measurement 2	3.67 ± 1.9	
	VD (mm)	Measurement 1	5.49 ± 2.1	<0.001
		Measurement 2	4.57 ± 1.8	

CEA: confidence ellipse area; TTL: total trajectory length; HD: horizontal deviation; VD: vertical deviation; *p*: significance level. Data are expressed as the arithmetic mean and standard deviation.

**Table 4:** Correlation between change in body balance and physical activity before arrival to the centre, BMI, and age.

Study conditions	Variables	Study group			Control group		
		Physical activity	BMI	Age	Physical activity	BMI	Age
Open eyes	CEA (mm^2^)	-0.12	-0.01	0.06	0.07	0.06	-0.03
	TTL (mm)	0.02	-0.07	0.11	0.28^∗^	-0.24	0.00
	HD (mm)	-0.18	0.12	0.08	-0.01	0.17	0.11
	VD (mm)	0.05	-0.07	0.00	-0.13	0.03	-0.10
Closed eyes	CEA (mm^2^)	-0.13	-0.04	-0.08	0.02	0.04	-0.05
	TTL (mm)	-0.09	-0.06	0.05	0.01	-0.12	0.10
	HD (mm)	-0.19	-0.08	0.00	-0.07	-0.01	-0.14
	VD (mm)	0.09	0.03	-0.13	-0.07	-0.01	-0.09
After the turns	CEA (mm^2^)	0.09	-0.21	-0.12	0.12	0.07	-0.05
	TTL (mm)	0.13	-0.03	0.09	0.18	-0.27^∗^	-0.10
	HD (mm)	0.13	-0.02	-0.10	0.03	0.05	-0.02
	VD (mm)	0.03	-0.23	-0.11	0.16	0.14	0.08

^∗^ *p* < 0.05; CEA: confidence ellipse area; TTL: total trajectory length; HD: horizontal deviation; VD: vertical deviation.

## Data Availability

The data used to support the findings of this study have not been deposited in the repository. The data used to support the findings of this study were not supplied under license. The data used to support the findings of this study are not currently under embargo while the research findings are not commercialized. The data used to support the findings of this study are restricted by the Bioethics Committee of the Jagiellonian University (number 122.6120.342.2016, 28 April 2017) and by the Scientific Research Team of the “Krzeszowice” Rehabilitation Centre (number 2/2017, 29 March 2017) in order to protect patient privacy. Data are available from Magdalena Pieniążek for researchers who meet the criteria for access to confidential data.
